# Ablation of Gut Microbiota Alleviates DON-Induced Neurobehavioral Abnormalities and Brain Damage in Mice

**DOI:** 10.3390/toxins17030144

**Published:** 2025-03-18

**Authors:** Yujing Cui, Samuel Kumi Okyere, Haoyue Guan, Zixuan Hua, Youtian Deng, Huidan Deng, Junliang Deng

**Affiliations:** 1 Key Laboratory of Animal Disease and Human Health of Sichuan Province, Sichuan Agricultural University, Chengdu 611130, China; yjchoi@163.com (Y.C.); samuel20okyere@gmail.com (S.K.O.); guanhaoyue08@163.com (H.G.); 13708235219@163.com (Z.H.); idyt@outlook.com (Y.D.); denghuidan@sicau.edu.cn (H.D.); 2 Department of Pharmaceutical Sciences, School of Medicine, Wayne State University, Detroit, MI 48201, USA

**Keywords:** deoxynivalenol, neurotoxicity, specific pathogen-free mice, behavioral studies, glial cells

## Abstract

Background: Deoxynivalenol (DON) poses a threat to animal and human health, particularly causing damage to the nervous system. Intestinal flora can regulate the nervous system through the gut–brain axis; however, there is currently a lack of evidence on the effect of changing the intestinal flora on the damage to the nervous system caused by DON. Therefore, this study aims to investigate the effect of gut microbiota ablation on neurotoxicity induced by exposure to deoxynivalenol. Methods: One hundred-twenty (120) specific pathogen-free (SPF) male C57BL/6j mice were randomly divided into four groups (control group, microbiota-uncleaned group + 5 mg/kg/BW DON, microbiota-cleared group, and microbiota-cleared group + 5 mg/kg/BW DON). The open field and Morris behavior tests were used to evaluate behavior changes after DON exposure. After 14 days of treatment, the mice were euthanized and brain tissues were collected for further analysis. Results: The tests showed that DON exposure led to anxiety and decreased learning ability in mice with no gut microbiota ablation. We also observed pathological changes including neuronal shrinkage, degeneration, and cortical edema in the mice with no microbiota ablation after DON exposure. In addition, the protein and mRNA levels of tight junction proteins and anti-inflammatory factors were decreased in the mice with no microbiota ablation after DON exposure compared with mice with ablated microbiota. Conclusions: We concluded that the presence of microbiota plays a key role in the neurotoxicity induced by DON; thus, ablation of the intestinal microbiota can effectively improve brain damage caused by DON.

## 1. Introduction

Deoxynivalenol (DON) is a single terminal fungal toxin produced by *Fusarium* under suitable temperature and humidity conditions and is widely present in grains and processed products such as corn, wheat, and bread [1,2]. DON has high stability and temperature tolerance and can still be detected in contaminated wheat after 4 years of storage [3]. Recent studies have found that DON can cause multi-organ damage in human and animal bodies, including growth inhibition, gastrointestinal injury, neurotoxicity, reproductive toxicity, immunotoxicity, carcinogenesis, and destruction of immune function [4,5,6,7]. Studies have shown that DON can trigger systemic toxicity through multiple pathways, such as activating oxidative stress, inducing mitochondrial apoptosis path, and disrupting the central nervous network [8,9,10]. Among them, research on neurotoxicity is increasing day by day as the brain is the highest component of the central system in the body. DON activates microglia [11], causes immune disorders, mediates neurodegenerative damage, and accelerates neurodegenerative diseases, leading to brain injury [12]. DON also activates oxidative stress [13], damages cell membranes and blood–brain barrier (BBB), triggers brain inflammation, and, ultimately, impairs the function of the entire central nervous system [14].

Numerous studies have shown that glial cells, especially astrocytes and microglia, play an irreplaceable core role in brain homeostasis, neural information transmission, neuronal survival, and neuroinflammation [15,16,17]. In addition, astrocytes and microglia serve as key players in brain injury diseases. Although existing information on DON-induced glial cell toxicity is still limited, previous studies have shown that DON can cause neuronal and brain damage by altering the state and function of glial cells [18,19]. DON also causes abnormal activation of the astrocytes to release various inflammatory factors [20].

Increase in gut permeability is associated with BBB damage as tight junction proteins are an important component of BBB. Behrens et al. [21] reported that DON can destroy the BBB by disrupting tight junctions.

The association between the gut microbiota and the microbiome–brain axis (MB Axis) has been a research hotspot in recent years. MB axis research mainly focuses on diseases such as Parkinson’s disease, multiple sclerosis, depression, autism, and obesity [22,23,24]. Through the study of these diseases, it has been found that gut microbiota plays an important role in regulating the nutritional metabolism and health of the body. Changes in microbiota abundance are closely related to gut function [25]. The regulation of the brain function by microbiota has also been confirmed and reported [26]. Ablation of gut microbiota has been reported to have many benefits in both humans and animals. A study by Sun et al. [27] reported that gut microbiota ablation alleviates obesity-induced hepatic steatosis and glucose intolerance by modulating bile acid metabolism. The study also suggested that the removal of the gut microbiota mainly elevated alternative bile acid synthesis in the liver and suppressed the microbial deconjugation and de-hydroxylation in the gut, leading to increased TβMCA and decreased secondary bile acids in the ileum, thus alleviating glucose intolerance. Another study also reported that ablation of the gut microbiota also alleviated high-methionine diet-induced hyperhomocysteinemia in mice [28]. Numerous studies have reported that DON can alter the abundance of microbiota in various animals. However, there is still limited research on the role of microbiota in neurotoxicity, especially DON-induced neurotoxicity. Therefore, after confirming the effects of DON on the gut and microbiota (unpublished), our research team intends to preliminarily explore whether microbiota ablation can alleviate or enhance DON-induced neurotoxicity. Therefore, this study was performed to investigate the effect of gut microbiota on DON-induced brain toxicity in mice to provide a research foundation for further mechanism analysis of MB axis in the future.

## 2. Results

### 2.1. Effects of Ablation of Intestinal Microbiota on Behavioral Changes in DON Fed Mice

#### 2.1.1. Results from Open Field Test

The movement trajectory diagram was drawn by the Shanghai Xinruan Animal Behavior Online Analysis System based on the mouse’s motion trajectory. The 40 × 40 activity area was divided into 16 squares, with the middle 4 squares called the central area, the 4 corner squares called the quadrilateral area, and the remaining side wall squares called the peripheral area. Time heatmap was drawn based on the time a mouse stayed at a certain location, with blue to red (0–10 s) indicating the duration of stay. An active heatmap was drawn based on the level of activity of each mouse at a certain location, with blue to red (0~1000 cm^2^/s) indicating the amount of activity over time.

The total horizontal movement distance refers to the total movement distance of a mouse within 300 s. The average speed is the ratio of the total distance to the time a mouse stays in an open field. The distance traveled to the central area refers to the distance traveled by the mouse in the central area. The visit time to the central area refers to the time the mouse is active in the central area, and the number of times the mouse enters the central area during the entire experimental period is also indicated.

Rearing is the number of times a mouse stands upright when both forelimbs are completely off the ground (i.e., both are lifted into the air (unsupported upright) or climb onto the box wall (supported upright) to maintain an upright position). Hair grooming is the behavior of mice licking or cleaning and grooming their facial, limb, trunk, and reproductive organs with their paws, with one licking or grooming session per session.

**Figure 1:**
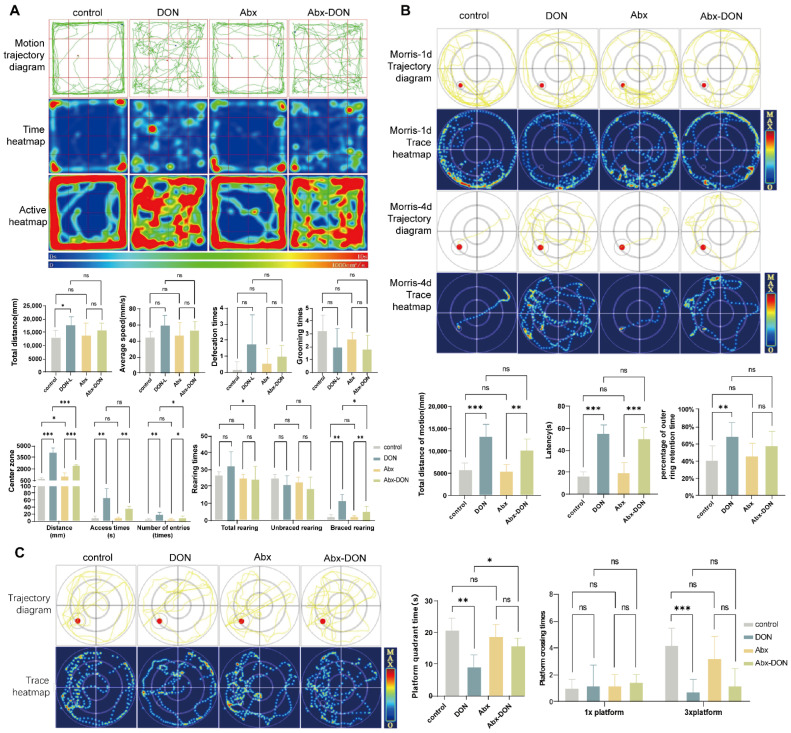
Effects of DON on behavioral tests in mice. (**A**) In the motion trajectory diagram of mine field experiment and open field test results, the red dot is the starting position and the blue dot is the ending position. Time heatmap blue to red: 0~10 s; activity heatmap blue to red: 0~1000 cm^2^/s) (the histogram shows the total distance of movement, average speed, central area activity data, defecation times, grooming times, and upright times). (**B**) The motion track diagram, heatmap (1 d and 4 d), and result bar diagram (4 d, in turn, total motion distance, latency, and percentage of outer ring retention time) of the water maze-concealed station experiment. (**C**) The motion track diagram, heatmap, and result bar diagram of the water maze spatial probe test (platform quadrant residence time and platform crossing times). All experimental data are expressed as “Mean ± SD”, ns indicates no difference, “*” *p* < 0.05 indicates significant difference, and “**” *p* < 0.01 and, “***” *p* < 0.001 indi-cates extremely significant difference.

As shown in Figure 1A, we observed that the control and Abx groups had similar movement trajectories, activity heatmaps, and time heatmaps. However, the movement trajectory map of the DON group was disorganized, and showed more central area activity with the longest stay time in the central area.

From the activity heatmap, the activity level of the Abx DON group in the central area was much higher than that of the control group and Abx group. The movement trajectory of ABX-DON was more inclined to the central area than that of the control group, whereas it was more inclined to the peripheral area than that of the DON group.

Compared with the control group, the total distance and number of times the DON group entered the central area were significantly higher (*p* < 0.05 and *p* < 0.01, respectively). The total distance covered by the mice in the box of the DON group was higher compared with the control group (*p* < 0.05). However, there was no difference in average speed, defecation time, and grooming time among all the groups (*p* > 0.05)

Furthermore, at the central area of the box, the total distance covered by the DON group was higher than that in the control group and the Abx DON group (*p* < 0.01). In addition, visit time by the DON group was higher compared with the control group (*p* < 0.001). The total distance and visit (access) time to the central area of the Abx DON group was higher than that in the Abx group (*p* < 0.01), and the number of times the mice in the Abx DON group entered the central area was also higher than that in the Abx group (*p* < 0.05). We also observed that the number of times the DON group entered the central area was significantly higher than that of the Abx DON group (*p* < 0.05).

The total rearing times in DON group were significantly higher than those in Abx-DON group (*p* < 0.05). The supported upright rearing (standing) values in the DON group were significantly higher than the control group (*p* < 0.01) and Abx DON group (*p* < 0.05); however, the upright values for the Abx DON group were significantly higher than the Abx group (*p* < 0.01). There was no difference in the unsupported upright rearing (standing) values among the groups.

#### 2.1.2. Results from Morris Test

- Hidden Platform results

The trajectory and route heatmaps of mice on the first day (Morris-1d) and last day (Morris-4d) of training were shown in Figure 1B. The maximum diameter of the circle represents the activity range of the water maze. According to the different diameters of the circles, this collection program divides the circles into three layers, with the outermost circle designated as the surrounding activity area and the rest as the central activity area. The red dot in the trajectory map represents the hidden platform, and the yellow line represents the automatically recognized mouse swimming route. The trace/route heatmap was collected based on the duration of mice activity in the mouse route map, with blue to red indicating the decreasing activity time at a certain location.

There are currently four different search strategies to evaluate mouse path maps: linear, directional, edge-based, and random. Linear strategy refers to the use of the line connecting the entry point of the experimental animal and the center of the platform as the central axis. If the distance between all the animal’s motion trajectories and the central axis does not exceed 15% of the radius, and the time spent in this area accounts for at least 70% of the total motion time, then the experimental animal’s motion is considered a linear strategy.

Trend-based strategy: Similar to linear strategy, the experimental animal’s movement is considered to be a trend-based strategy if the distance between all the animal’s movement trajectories and the center of the platform is not more than 50% of the radius, and the time spent in this area accounts for at least 70% of the total movement time, with the line connecting the experimental animal’s entry point into the water and the center of the platform as the axis.

Edge-based strategy: Taking the center of the experimental animal’s movement area as the center and taking 75% of the radius as a circle, if the animal is active outside the circle for more than 70% of the time, the experimental animal’s movement at this time is considered an edge-based strategy.

Random: If the movement strategy of the experimental animal is different from the above three, it is considered that the animal’s movement is a random strategy.

Due to the marginalization and circumnavigation of rodent swimming, it was clearly observed in Morris-1d that most of the mice in each group move within the surrounding range, swim close to the edge of the water basin, and adopt an edge-based search strategy. During Morris-4d, it was observed that the control group and Abx group mice could quickly find the platform by memorizing the patterns on the edge of the water basin, using a linear search strategy, while the DON group and Abx DON used a directional or random search strategy, requiring a relatively long path to find the platform or still unable to find it. The total distance of motion in the DON group was significantly higher than that of the control group (*p* < 0.01) after Morris-4d. In addition, the total distance of motion of the Abx DON group was significantly higher than the Abx group (*p* < 0.01); however, there was no difference in the total distance of motion between the DON and Abx DON groups (*p* > 0.05).

The latency period refers to the time required for experimental animals to successfully find the platform for the first time after entering the water. The latency period of the DON group was significantly higher than that of the control group (*p* < 0.01). The latency period of the Abx DON group was significantly higher than the Abx group (*p* < 0.01). There was no difference in the latency period between the DON group and the Abx DON group (*p* > 0.05).

The percentage of outer ring retention time in the surrounding activity area is the sum of the time the experimental animals spent in the area around the pool, as a percentage of the total activity time. The percentage of outer ring retention time in the surrounding activity area of the DON group was significantly higher than that of the control group (*p* < 0.01), whereas there was no difference in the percentage of outer ring retention time in the surrounding activity area among the other groups (*p* > 0.05).

- Spatial probe test results

In the spatial exploration experiment (Figure 1C), the yellow is the motion trajectory, the red dot is the platform, the original platform size is recorded as 1×, the three times the radius of the original platform is recorded as 3×, and the small circle outside the red dot is the 3× platform area.

The platform quadrant time refers to the time the experimental animal is active in the quadrant where the platform is located.

The number of crossings refers to the number of times the experimental animal crosses the original platform within a certain period of time after the platform is removed. One entry and exit of the platform is recorded as one crossing.

We observed that the platform quadrant time of the control group was significantly higher than that of the DON group (*p* < 0.01) and the platform quadrant time of the DON group was higher than the Abx-DON group (*p* < 0.05). However, there was no difference in the platform quadrant time between the Abx group and the Abx-DON group (*p* > 0.05). There was no significant difference in the number of mouse 1× platform crossings (*p* > 0.05) among the groups. The number of mice 3× platform crossings in the control group was significantly higher than in the DON group (*p* < 0.01); however, there was no difference in the number of mice 3× platform crossings between the Abx and the Abx-DON groups and the DON and Abx-DON groups (*p* > 0.05).

### 2.2. Effects of Intestinal Ablation on Brain Specific Gravity

**Figure 2:**
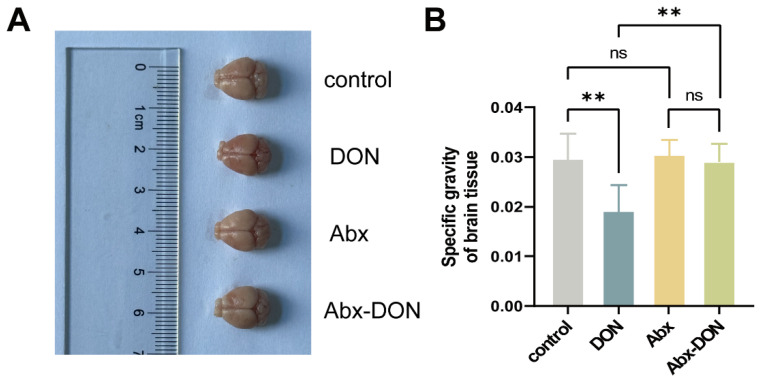
Brain sampling (**A**) and specific gravity of brain tissue (**B**). All experimental data are expressed as “Mean ± SD”, ns indicates no difference and “**” *p* < 0.01 indicates significant difference.

Figure 2A shows various brain tissues after the removal of the olfactory bulb. The results (Figure 2B) showed that compared with the control group, the brain density of the DON group was significantly reduced (*p* < 0.01); however, there was no difference in brain density between the Abx and the Abx DON groups (*p* > 0.05). Moreover, compared with the Abx DON group, the brain density in the DON group was significantly reduced (*p* < 0.01).

### 2.3. Effects of Interstinal Ablation on Pathology of Brain Tissue of Mice Fed DON

#### 2.3.1. H&E Staining and Nissl Staining Results

**Figure 3:**
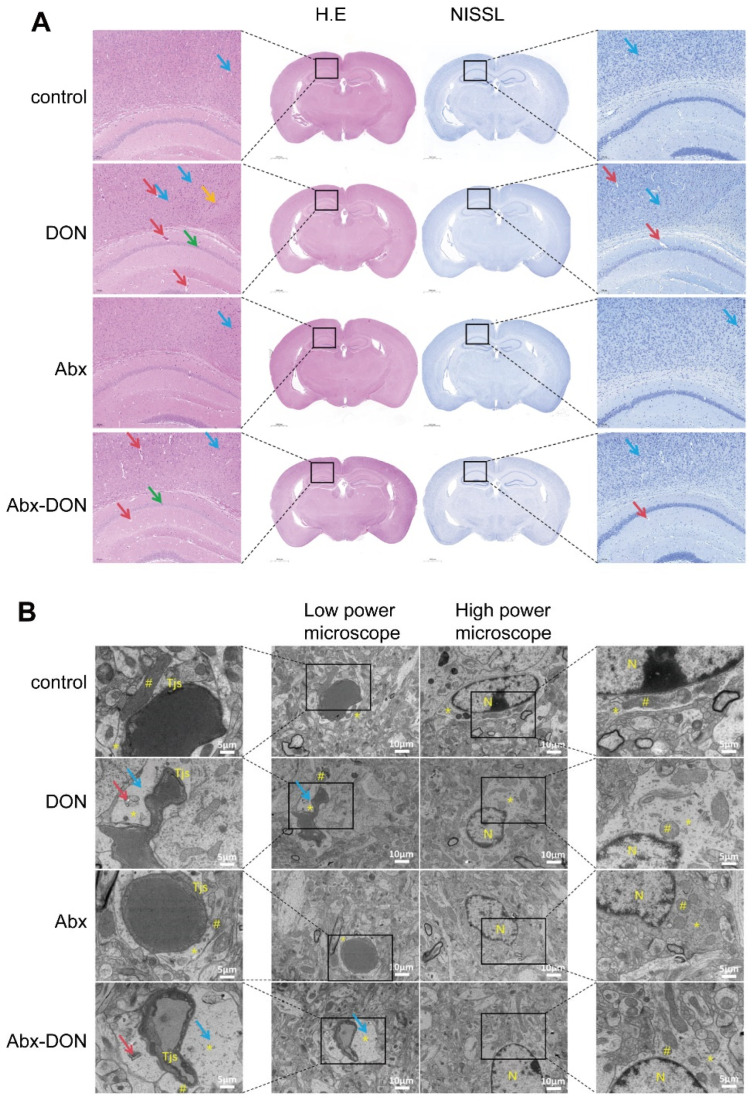
Effects of DON on brain histopathology in mice. (**A**) The coronal H.E staining and Nissl staining of the brain were HE 1.3x (Bar = 1000 μm), HE 8x (Bar = 100 μm), Nissl 1.3x (Bar = 1000 μm), Nissl 8x (Bar = 100 μm) from left to right. Blue arrow: neuronal shrinkage degeneration in the cerebral cortex; green arrow: neuronal shrinkage degeneration in the hippocampus; red arrow: mild to moderate edema around blood vessels; yellow arrow: local nerve tissue degeneration, loose structure. (**B**) Neuronal ultrastructural pathology, from top to bottom: control group, DON group, Abx group, ABX-DON group, low power bar = 10 μm, high power bar = 5 μm. Yellow marks indicate the structure: #: mitochondria, *: glial process, Tjs: tight junction, N: glial cell nucleus; red arrow: autophagy; blue arrow: glial swelling.

The results from the H&E and Nissl staining were shown in Figure 3A. The 1.3× images of HE and Nissl staining in each group showed that the brain structure was intact; the cerebral cortex, subcortical nuclei, and central sulcus had normal morphology and were composed of a large number of neuronal cell bodies, glial cells, and nerve fibers. The boundaries of the lateral ventricles were clear, the hippocampus and its dentate gyrus were clearly visible, and there were neurons rich in large cell bodies. The 8× magnification of the sections of each group revealed that there were no obvious pathological changes in the microglia and astrocytes, hippocampal neurons, and cerebellum of the control and Abx groups; however, only a few neurons were pyknotic (blue arrows). In the DON group, some neurons were pyknotic in the cerebral cortex and hippocampus (green arrows), and there was mild to moderate edema around the local blood vessels in the cerebral cortex and hippocampus (red arrows). Neural tissue degeneration and loose structure appeared in some parts of the brain (yellow arrows). In the Abx-DON group, a small number of neurons were pyknotic and degenerated in the cerebral cortex and hippocampus, and there was mild edema around the local blood vessels in the cerebral cortex and hippocampus. The severity of the lesions in each group from mild to severe was as follows:

control group ≈ Abx group < Abx-DON group < DON group

#### 2.3.2. Ablation of Intestinal Microbiota Alleviate Neuronal Ultrastructure Pathological Damage in DON Fed Mice

Morphological description and localization analysis were performed on ultra-thin sections of each group using electron microscopy combined with relevant histological and cytological characteristics. Two representative fields of view were selected for each group, and two images were captured at low magnification (bar = 10 μm) and high magnification (bar = 5 μm), respectively. The results are shown in Figure 3B. The low magnification field can observe cerebral cortical capillaries and tight junctions, as well as glial cells, glial cell nuclei, and glial cells, whereas the high magnification field is an enlargement of the selected area in the low magnification field. Under electron microscopy, the cerebral cortex capillary structures of the control group and Abx group were normal, with tight connections and good occlusion. There were no abnormalities in the peripheral glial cell protrusions, and the nuclei and mitochondria of glial cells were normal. The morphological structure was intact. In contrast, the cerebral cortex capillary structures of the DON group were abnormal, and normal nuclei and mitochondria of glial cells could be observed in the DON group; however, the peripheral glial cell protrusions showed mild to moderate swelling (blue arrow) and autophagy (red arrow). The lesions in the Abx DON group were similar to those in the DON group, but the swelling of the peripheral glial cell protrusions was more pronounced. The severity of the lesions in each group, from mild to severe, was as follows:

control group ≈ Abx group < DON group < Abx DON group.

### 2.4. IF Detection of DON Expression in Brain Tight Junctions, Glial Cells, and Neurons

**Figure 4:**
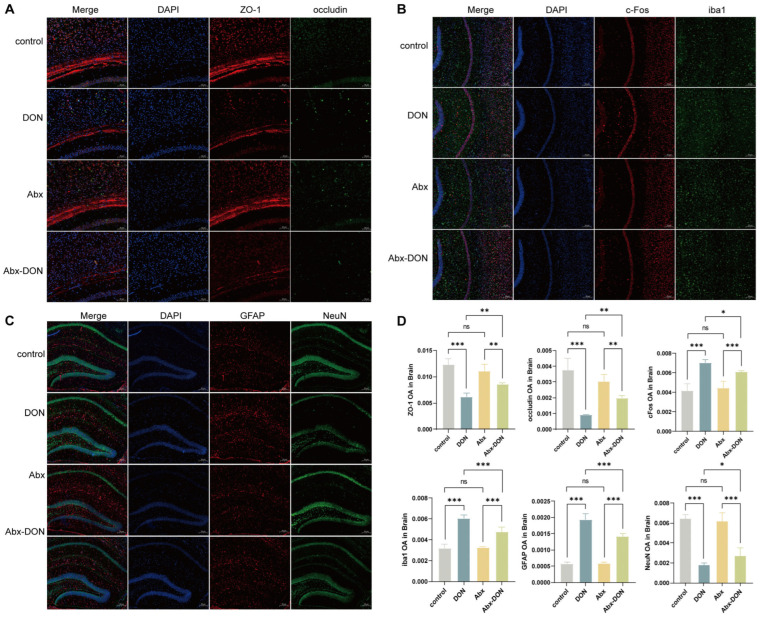
Location and expression of DON on glial cells, neurons, and tight connections. (**A**) Tight junction protein ZO-1 + occludin; (**B**) c-FOS + iba1; (**C**) GAFP + NeuN; (**D**) ZO-1/occludin/c-FOS/iba1/GAFP/NeuN OA in brain. All experimental data are expressed as “Mean ± SD”, ns indicates no difference, “*” *p* < 0.05 indicates significant difference, and “**” *p* < 0.01 and, “***” *p* < 0.001 indi-cates extremely significant difference.

The IF results (Figure 4A,D) showed that compared with the control group, the expression levels of ZO-1 and occludin proteins in the DON group were significantly reduced (*p* < 0.05). Compared with the Abx group, the expression levels of ZO-1 and occludin proteins in the Abx DON group were also significantly reduced (*p* < 0.05). However, the expression levels of ZO-1 and occludin proteins in the DON group were significantly lower than those in the Abx DON group (*p* < 0.05), indicating that the level of tight junction proteins decreased more in the presence of bacteria. The IF results showed (Figure 4B–D) that DON significantly increased the protein expression levels of c-FOS, iba1, and GFAP compared with the control and Abx DON groups (*p* < 0.05). On the contrary, DON significantly reduced the expression level of NeuN protein compared with the control and the Abx DON groups (*p* < 0.05).

### 2.5. Western Blot Detection of the Effects of DON Expression on Inflammatory Cytokines in the Brain

**Figure 5:**
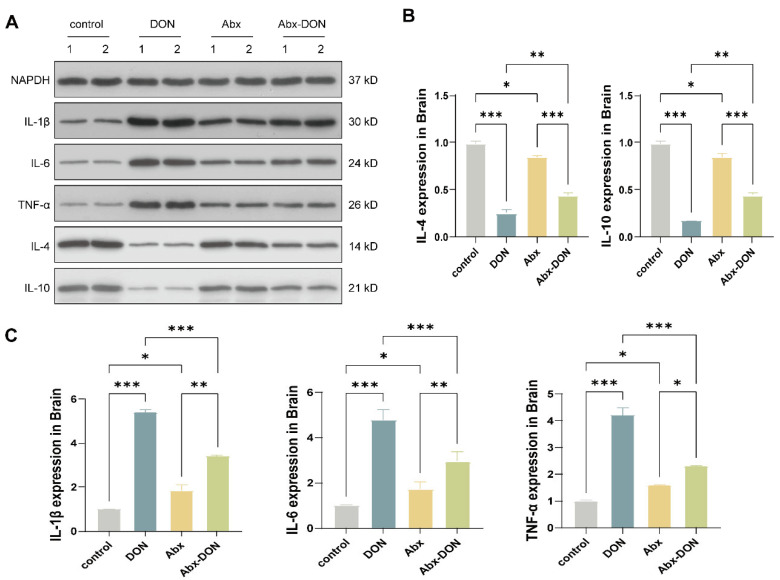
Western blot results of DON on encephalitis factors. (**A**) W strip; (**B**) results of normalization of anti-inflammatory factor data (IL-4, IL-10); (**C**) results of normalization of pro-inflammatory factor data (IL-1β, IL-6, TNF-α). All experimental data are expressed as “Mean ± SD”, ns indicates no difference, “*” *p* < 0.05 indicates significant difference, and “**” *p* < 0.01 and, “***” *p* < 0.001 indi-cates extremely significant difference.

The WB results of cytokine protein expression is shown in Figure 5A. The quantification results showed that compared with the control group, the protein levels of pro-inflammatory factors (IL-1β, IL-6, TNF-α) in the DON group were significantly increased (*p* < 0.05, Figure 5C), and compared with the Abx group, the protein levels of pro-inflammatory factors (IL-1β, IL-6, TNF-α) in the Abx DON group were also significantly increased (*p* < 0.05). In addition, we observed that compared with the Abx DON group, the DON group had elevated levels of IL-1β, IL-6, and TNF-α in the brain (*p* < 0.05). The protein expression levels of anti-inflammatory cytokines (IL-4, IL-10, Figure 5B) in the DON group were significantly lower than those in the control group (*p* < 0.05). Similarly, the protein expression levels of anti-inflammatory cytokines in the Abx DON group were significantly lower than those in the Abx group (*p* < 0.05), and the protein expression levels of anti-inflammatory cytokines in the Abx DON group were significantly higher than those in the control group (*p* < 0.05).

### 2.6. RT qPCR Detection of the Expression Levels of Related Proteins at the mRNA Level

**Figure 6:**
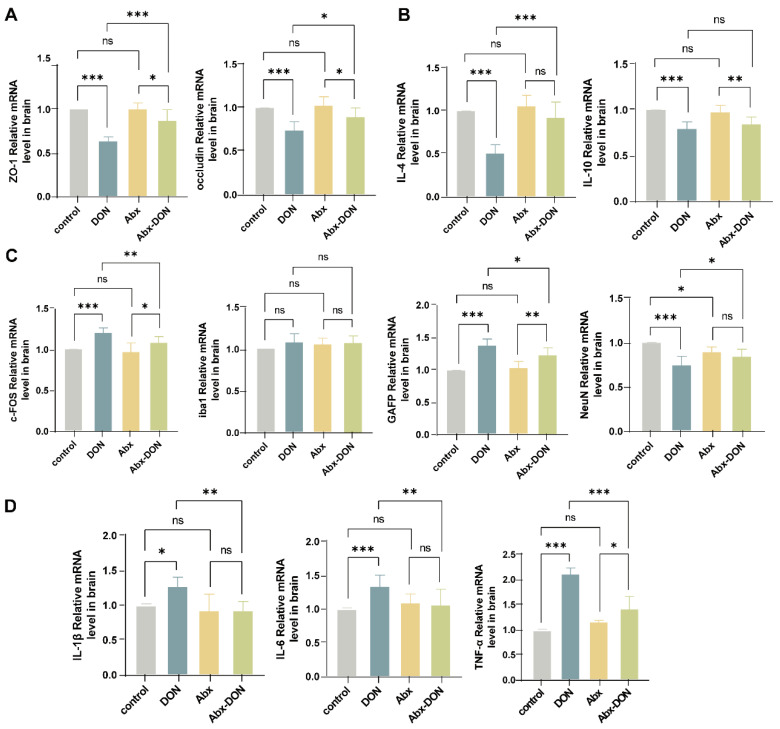
Effects of DON on mRNA levels of brain compact junctions, glial cell markers, neuronal markers, and inflammatory factors. (**A**) mRNA levels of tight junction protein-related genes (ZO-1 and occludin; (**B**) mRNA levels of anti-inflammatory factor related genes (IL-4 and IL-10); (**C**) mRNA levels of glial and neuronal markers (c-FOS, iba1, GFAP and NeuN); (**D**) mRNA levels of proinflammatory factor-related genes (IL-1β, IL-6, TNF-α). All experimental data are expressed as “Mean ± SD”, ns indicates no difference, “*” *p* < 0.05 in-dicates significant difference, and “**” *p* < 0.01 and, “***” *p* < 0.001 indi-cates extremely significant difference.

The mRNA levels of the mouse brain tight junction protein were detected (Figure 6A), and it was found that the mRNA levels of ZO-1 and occludin in the DON group were significantly lower than those in the control and Abx DON groups (*p* < 0.05), whereas the Abx DON group was also lower than the Abx group (*p* < 0.05). The mRNA level of brain glial cell markers and neuronal marker proteins (Figure 6C) showed that compared with the control group, the mRNA level expression of c-FOS and GFAP in the DON group were significantly increased (*p* < 0.05); however, there was no difference in the mRNA level expression of iba1 among all the groups (*p* > 0.05). The mRNA expression level of NeuN was significantly decreased in the DON compared with the control (*p* < 0.05). In addition, compared with the DON group, the mRNA level expression of c-FOS and GFAP in the Abx DON group was significantly decreased (*p* < 0.05), whereas the mRNA level expression of NeuN was significantly increased (*p* < 0.05). For the mRNA levels of inflammatory factors (IL-1β, IL-6, TNF-α) in mouse brain tissue (Figure 6B,D), we observed that compared with the control group, the mRNA levels of pro-inflammatory factors (IL-1β, IL-6, TNF-α) in the DON group were significantly increased (*p* < 0.05) and, also, significantly higher than those in the Abx DON group (*p* < 0.05). The level of anti-inflammatory factor IL-4 in the DON group was significantly lower than the control and Abx DON groups (*p* < 0.05). Similarly, the IL-10 levels in the DON group were significantly lower than the control group; however, there was no difference compared with the Abx DON group (*p* > 0.05).

## 3. Discussion

The brain is an important organ of life and the main signal transduction site of the central nervous system. It is responsible for detecting, transmitting, integrating, and responding to signals from both internal and external environments. In addition to participating in carbohydrate metabolism, fiber degradation, and immune response, the gut microbiota also participates in regulating the host’s neurophysiological functions, such as neural signal transduction, synaptic plasticity, neurotransmitter synthesis, and transport. Together, these two components form the MGB axis [29,30,31,32]. DON causes neurological symptoms and behavioral changes in animals [33], and the role of microbiota in animal behavior has also been reported. Furthermore, the association between changes in the structure of gut microbiota and host behavior has been established in the literature. One of the most commonly used behavioral tests in rodents is the open field test. This test can be used to evaluate their spontaneous activity, exploratory behavior, and anxiety and depression states [33]. The frequency of defecation and grooming can be used to evaluate an animal’s stress and anxiety state in a new environment. Moreover, upright posture can also reflect an animal’s exploration and curiosity about new environments. When an animal is in a depressed state, its level of curiosity decreases and the number of upright postures decreases. In our study, we found that mice exposed to DON exhibited a “less wall sticking” state, explored more non-central areas, and stayed in the four corner areas for shorter periods of time. At the same time, the total distance of movement and defecation, grooming behavior, and upright behavior increased, indicating that the animals exhibited a more anxious state, which is consistent with the results from a study that exposed mice to 100 μg/kg body weight (bw)/day of DON [34]. In addition, the anxiety state of mice with ablated microbiota was lower than that of mice directly exposed to DON.

The water maze is a classic experiment used to evaluate the learning, memory, and spatial cognitive abilities of animals [35]. Currently, it is mainly divided into concealed platform experiments and spatial exploration experiments. In general, normal mice can quickly and accurately find the platform after 2–3 days of training. However, when the learning and memory abilities of mice are impaired, they often cannot find the platform within the specified time [36]. In our study, we found that the search strategy of mice not exposed to DON changed from “edge type → trend type → linear type”, while mice exposed to DON showed an “edge type → random type” search strategy, showing poorer learning and memory abilities. This indicates that exposure to DON leads to a decrease in memory ability of mice, but this was not observed in the mice with ablated gut microbiota. Gut microbiota ablation may improve the learning and memory abilities of mice. Although there is limited research on the neurotoxicity caused by DON-induced changes in gut microbiota, a study by Diao et al. [37], which focused on the effects of other toxic substances (silicon dioxide nanoparticles) on animal behavior, showed that spatial learning, memory impairment, and motor inhibition may be significantly related to specific chemicals produced by gut microbiota, providing new insights into how microbiota affects toxin-induced nerve damage.

Under normal circumstances, the weight of brain tissue is affected by animal growth and development. When we further calculated the brain density of mice without microbiota ablation, we found that their brain density was significantly reduced, which is consistent with the results of Ren et al. [38]. We hypothesize that DON affects brain development; however, the presence of gut microbiota and their metabolites can exacerbate the brain damage through MB axis, and this may be the reason for more anxiety and reduced learning ability in mice without microbiota ablation.

The complete brain structure is the key to the formation of normal brain tissue; however, our experimental results showed that mice exposed to DON exhibited lesions characterized by neuronal shrinkage degeneration, neural tissue degeneration, mild edema around local blood vessels in the cerebral cortex and hippocampus, and mild to moderate swelling of glial cell processes. This is consistent with the study by Wang et al. [8] who observed cytoplasmic swelling in hippocampal cells after exposing pigs to 1.3 mg/kg/BW DON. However, the brain damage in mice with microbiota ablation was significantly milder than that in mice without microbiota ablation, further indicating that ablation of microbiota can alleviate the pathological damage of DON to the brain.

BBB is formed by the tight binding of endothelial cells and glial cells, which can control the entry of molecules from plasma into cerebrospinal fluid (CSF) [39]. BBB can regulate body fluids and protect the brain [40]. In vivo studies have shown that DON can be detected in the animal brain within 2–60 min after ingestion (depending on the animal species), indicating that the integrity of the BBB is an important indicator for evaluating DON-induced brain injury [41]. Tight junction proteins construct the structure of intercellular connections, maintain cell barrier function, and regulate intercellular permeability. In this study, although the protein and gene levels of ZO-1 and occludin were reduced in mice with gut microbiota clearance exposed to DON, they were higher than in those directly exposed to DON, indicating that the integrity of the BBB was disrupted. However, the clearance of the gut microbiota alleviated this damage. Studies have shown that short chain fatty acids (SCFAs), metabolic products of intestinal bacteria, can regulate BBB permeability [42]. Although the specific regulatory mechanism remains to be elucidated, the ablation of the microbiota may alleviate this damage.

This study also found that DON increased the protein and gene levels of c-FOS, iba1, and GFAP in the mouse brain while decreasing the protein and gene levels of NeuN. This is consistent with the results of Stéphane Gaigé’s exposure test to porcine [43] and Sakshi Mishra’s exposure test to DON in mice [44]. C-Fos belongs to the immediate early gene (IEG), which is a nuclear protein encoded by early response genes that can be enriched and expressed in neurons. Under normal physiological conditions, it is in a low expression or quiescent state and is difficult to detect. However, when the brain is attacked or damaged by toxins, the c-FOS gene quickly responds to high expression as an immediate early response gene. Stefaniuk et al. [45] found after behavioral training that the formation of long-term memory requires the participation of c-fos. When the animal’s learning curve approaches the plateau, the expression of c-fos decreases by 44%. Therefore, the increase in c-FOS caused by DON may also be the reason for the decreased memory in mice in this experiment. NeuN is a specific marker for differentiated mature neurons. The results of this study indicate that DON leads to a decrease in mature neurons, but ablation of the gut microbiota alleviates this damage. Although there is currently limited literature directly studying the relationship between NeuN and gut microbiota, previous studies have shown that changes in gut microbiota abundance affected the expression of NeuN [46].

The elevation of GFAP and iba1 suggests activation of astrocytes and microglia, which typically play important roles in immune regulation, including sensing harmful stimuli in the environment, swallowing debris and abnormal proteins, digesting apoptotic neurons, and performing antigen delivery function [18]. However, the damage to astrocytes and microglia was improved after ablating the microbiota, which may be due to DON inhibiting the uptake of L-glutamate produced by the microbiota by astrocytes [47]. Activated astrocytes and microglia usually have the ability to release soluble neuroinflammatory factors, and their immune response is considered a necessary condition for neuroinflammation [48,49]. A study by Girardet et al. [50] reported that DON could upregulate IL-1β. The expression of IL-6 and TNF-α pro-inflammatory cytokines induces central nervous system inflammation, which is consistent with the results of this study. Interestingly, intestinal inflammation caused by local infections or toxic substances can induce immune cells in the intestine to release pro-inflammatory cytokines, such as IL-1β, IL-6, and TNF-α, which can damage the tight junctions of the intestinal epithelium, thereby destroying the integrity of the BBB and intestinal mechanical barrier. The increase in intestinal permeability and the destruction of the central nervous system barrier provide a channel for intestinal derived molecules to reach the brain parenchyma, activate local immune cells in the brain, and trigger neuroinflammation [51]. The microbiota transmits these cytokines to the brain through infectious and regulatory factors. When the microbiota is dysfunctional, the substances transmitted to the brain promote chronic inflammatory reactions, increase oxidative stress, and internal energy imbalance [52]. A study showed that DON could significantly elevate the hypothalamic mRNA levels of proinflammatory cytokines (IL-1β, TNF-α, and IL-6) [53]. In addition, DON enhances mRNA production of IL-1β, IL-6, and TNF-α in the hypothalamus and in the DVC, two central structures that act as gateways for circulating chemicals and are strongly associated with food intake regulation [54]. DON has been proven to induce intestinal immunity and damage intestinal permeability, and in this experiment, the changes in pro-inflammatory and anti-inflammatory factors in the brain of mice with ablated microbiota were lower than those in mice without ablated microbiota, further indicating that microbiota may play an important role in DON-induced brain inflammation.

The bidirectional interaction between the brain and gut has been scientifically studied, but the role of gut microbiota (including symbiotic and pathogenic microorganisms) and their metabolites has only been gradually recognized in the past decade [55]. Therefore, gut microbiota is considered a potential factor in regulating the gut–brain axis, while microbial metabolites may be important regulatory factors [56]. In recent years, there have been few studies on the detoxification of DON by gut microbiota. Although it has been confirmed that natural gut bacteria do not have the ability to detoxify DON, in vitro studies have demonstrated the ability of gut microbiota to clear DON [57]. It has also been reported that the microbiota in the large intestine of broiler chickens can convert DON into the less toxic de-epoxidized DON through cyclooxygenase [58]. Based on the results of this study, it is possible to clarify that the ablation of the gut microbiota may be a new DON detoxification method. The main limitation of this study is the absence of the mode of action associated with ablation of gut microbiota on reverting DON toxicity, which is going to be our future project.

## 4. Conclusions

Ablation of the gut microbiota can effectively alleviate the brain damage caused by DON exposure and may be related to the microbiota–brain axis, suggesting that the microbiota may act on the brain along the microbiota–brain axis through its metabolites, which is going to be our next research direction. This study will provide strong support for the role of microbiota in the neurotoxicity caused by DON and provide research ideas for the development of DON biological detoxification agents.

## 5. Materials and Methods

### 5.1. Experimental Material and Design

One hundred-twenty (120) SPF male C57BL/6j mice (6–8 weeks old; weighing 20 ± 2 g) were purchased from the SiBeiFu (Beijing, China) and adaptively fed for one week. Vancomycin and metronidazole were purchased from Shanghai Yuanye Biotechnology Co., Ltd. (Shanghai, China), whereas neomycin sulfate and ampicillin were purchased from Shanghai Yien Chemical Technology Co., Ltd. (Shanghai, China). DON was purchased from Yujing Technology Co., Ltd. (Shanghai, China) (purity ≥ 99.6%).

**Figure 7:**
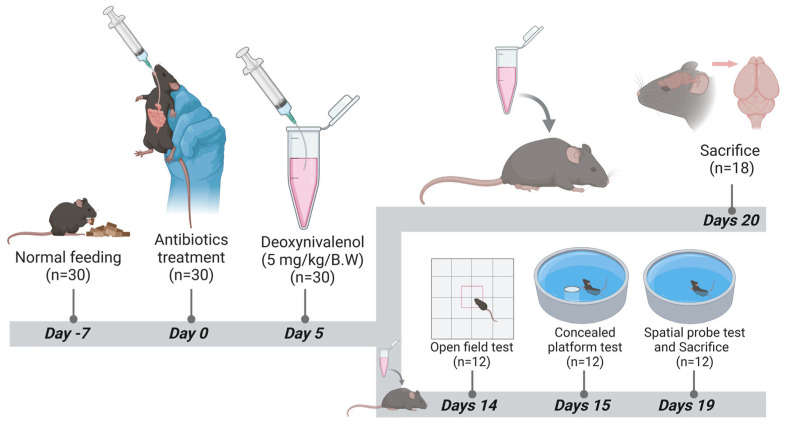
Experimental groups and their treatments.

Using the random number method, 120 mice were divided into four groups with 30 mice in each group. The groups and their respective treatments are shown in Figure 7.

Mice were kept in clean cages at room temperature and humidity of 40−70%. Mice were given free access to feed, drinking water, and natural light. The cages were cleaned and disinfected daily. On the 13th day of the experiment, 48 mice were randomly selected from each group for animal behavior experiments.

### 5.2. Antibiotics Preparation and Treatment

Four antibiotics were used to create the pseudo sterile mouse model following the procedure of Gong et al. [59]. The antibiotic solution was prepared by dissolving vancomycin (100 mg/kg BW), neomycin sulfate (200 mg/kg BW), metronidazole (200 mg/kg BW), and ampicillin (200 mg/kg BW) in 1X PBS solution and then administered orally every morning at 8:00 a.m. for 5 days.

### 5.3. DON Preparation and Administration

The dosage and timing of DON were based on pre-experimental and previous research results of our research group. DON was dissolved in UP water and orally administered at 5 mg/kg/BW every morning at 8:00 a.m.

### 5.4. Behavioral Experiment

#### 5.4.1. Open Field Test (OFT)

During animal rearing, in addition to gavage, mice were stroked for 2–5 min every day to familiarize them with the experimenters. After 8 days of the experiment, xx mice were transferred into the laboratory 3 h before the experiment to fully adapt to the spatial environment. Before the test, the open field box (40 cm × 40 cm × 50 cm) was disinfected with 75% ethanol and dried to ensure cleanliness and odorlessness. All groups of mice completed the test between 8:00 and 12:00 in the morning. Each mouse was tested once for 5 min each time. After each test, the excrement of the previous mouse was removed and the odor was removed with 75% ethanol and dried before the next mouse was tested. Before the experiment, the open field box was placed in the center of the laboratory floor, and the height of the camera was adjusted to ensure that all areas inside the box can be recorded. During the experiment, the mice were removed from their cages via their tail (one-third) and were placed gently and quickly in the center of the open field box with their back facing the experimenter. At the same time, the video capture system was activated to record the activity of the mice in the open field for 5 min; then, the mice were removed and placed back into their feeding cage.

After the experiment was completed, the collected videos were uploaded to the Shanghai Xinruan Animal Behavior Online Analysis System for analysis, and motion indicators, including total horizontal distance, average speed, number of times the central area was entered, central area exercise time, and percentage, were obtained. Behavior parameters including upright frequency (divided into unsupported upright and supported upright), defecation frequency, grooming frequency, mouse motion roadmap, motion time heatmap, and activity heatmap were calculated.

#### 5.4.2. Morris

- Concealed platform test

Twenty-four (24) hours after the end of the open field experiment, the mice were placed in a white water pool with a diameter of 1.2 m and a height of 0.5 m. The water temperature was maintained at around 23 °C, and four quadrants I–IV (northwest, northeast, southeast, southwest) were set up. Each quadrant had a reference object on the pool wall about 1 cm below the liquid level with a platform diameter of 0.06 m and a height of 0.2 m. The mice were placed in quadrant II, and the camera was placed directly above the pool.

The experiment lasted for 4 days, with training conducted 4 times a day at fixed time intervals. At the beginning of each day, mice were placed facing the pool wall from one of the four quadrants into the water. Each experimental mouse was allowed to swim for 0–60 s each time to find the hidden platform. If the mouse successfully found the platform, the mouse was allowed to rest on the platform for 15 s; however, if the mouse could not successfully locate the platform within 60 s, the mouse was manually guided to rest on the platform for 15 s to ensure that each mouse had equal time to observe and obtain spatial information in each experiment. The TaiMeng WMT-100 Morris water maze video analysis system was used to automatically record the total distance of the mouse movement and find the time (latency) of the platform and the swimming path map.

- Spatial probe test

On the 5th day, the platform was removed and one entry point was selected for the mice to enter the water. All the mice were strictly allowed to pass the same entry point, and then, the swimming path of the mice within 90 s, the time they stayed in the original platform quadrant, and the number of times they crossed the platform and its surroundings (3 times) were recorded to observe their spatial positioning ability.

### 5.5. Brain Specific Gravity Measurement

After the 19th day of the experiment, the remaining 72 mice (those mice not used for conducting behavioral experiments) in various groups underwent fasting for 4 h. Twelve mice were randomly selected from each group, weighed, and subjected to the following procedures: decapitation, immediate dissection, separation of skull and olfactory bulb, and a complete removal of the brain. After the brain was completely removed, it was washed with pre-cooled PBS, dried with filter paper, and the olfactory bulb was removed. The weight of the brain was measured and the brain specific gravity was calculated as follows:

Brain specific gravity = brain weight/body weight.

### 5.6. Sample Collection and Preservation

After weighing the brain, the experiment proceeded as follows:

a. Six individuals were randomly selected from each group and their entire brains were sliced by pre-cooling with 10% paraformaldehyde.

b. Three animals were randomly selected from each of the remaining nine mice and 2 mm slices were cut from the coastal horse crest with a blade. The slices were then placed in a pre-cooled electron microscope solution and viewed under an electron microscope.

c. The remaining brain tissue from (b) 3 mice and the remaining 6 mice brains (about 50 mg) were rapidly frozen in liquid nitrogen for later use.

### 5.7. Pathology of Brain Tissue

#### 5.7.1. HE Staining of the Brain

Mouse brain tissue was fixed in a 4% paraformaldehyde solution for 36 h, embedded in paraffin blocks, and a transverse sectioning at a thickness of 4 μm was performed using a conventional rotary slicer(Leica, RM2235, Nußloch, Germany). After dewaxing, the sections were stained with hematoxylin for 3 min and eosin for 5 min for morphological analysis. 3DHISTECH (Budapest, Hungary) Pannoramic SCAN digital slice scanner and CaseViewer 2.3 software were used to observe and analyze pathological changes in the brain and capture low or high magnification images.

#### 5.7.2. Nissl Staining

First, the slices were sequentially placed into xylene I for 20 min, xylene II for 20 min, anhydrous ethanol I for 5 min, anhydrous ethanol II for 5 min, and 75% alcohol for 5 min. After washing with tap water to dewax, the tissue slices were then immersed in Nissl staining solution for 45 min. The slices were washed with water to stop the reaction and control the degree of differentiation under a microscope. After washing with tap water, the slices were placed in an oven to dry. The slices were cleaned in xylene for 5 min and sealed with neutral gum. The 3DHISTECH (Hungary) Pannoramic SCAN digital slice scanner was used to capture images and store them for future use. The images of each group were analyzed using the CaseViewer software.

#### 5.7.3. Neuronal Ultrastructure Pathology

Tissue from the same part of the cerebral cortex was taken, pre-fixed with 2% paraformaldehyde-2.5% glutaraldehyde, and post-fixed with 1% osmium tetroxide buffer solution. The parameters for specimen dehydration, resin immersion, and polymerizing embedding were as follows: 30% ethanol for 10 min, 50% ethanol for 10 min, 70% ethanol for 15 min, 90% ethanol for 15 min, anhydrous ethanol for 15 min, acetone for 15 min twice, resin-acetone 1:3 mixture for 30 min, resin-acetone 1:1 mixture for 30 min, resin-acetone 3:1 mixture for 1 h, resin embedding solution at 37 °C overnight, new embedding solution at 40 °C for 8 h, and polymerization at 60 °C for 48 h.

The embedded blocks were semi-thinly sectioned at a thickness of 0.8 μm, stained with toluidine blue, and observed and positioned under a light microscope, and the same layer area where the cortical neurons were located was selected. Ultra-thin sections were made at a thickness of 90 nm and mounted on a 200-mesh Fanghua membrane copper grid. Conventional uranyl acetate-lead citrate electron staining was performed for 15 min and 5 min, respectively, and then, the samples were dried after staining. The ultra-thin section grid was imaged at 80 kV on a Hitachi HT7800 transmission electron microscope(Hitachi High-Tech Corporation, Tokyo, Japan) to analyze its pathological changes and collect images.

### 5.8. Immunofluorescence Detection of DON Localization and Expression in Brain Glial Cells, Neurons, and Tight Junctions

The prepared brain paraffin blocks were cut into 4 µm thick slices. Two discontinuous slices were made for each sample and then dewaxed in water followed by antigen repair, endogenous peroxidase blocking, and blocking with 5% goat serum. The primary antibody was added and incubated in a humidified box at 4 °C overnight, and the secondary antibody was added and incubated at 37° in the dark for 40 min. The cell nucleus was counterstained with DAPI and incubated at room temperature in the dark for 10 min. After sealing with anti-fluorescence quenching mounting medium, the slices were placed under a fluorescence microscope for observation and image collection. The changes in neurons and glial cells were detected by GFAP (GB12096, Wuhan Servicebio Technology Co., Ltd., Wuhan, China), NeuN (DF6145, Proteintech Group, Inc., Chicago, United States), c-FOS (GB12096, Wuhan Servicebio Technology Co., Ltd., Wuhan, China) and iba1 (GB113502, Wuhan Servicebio Technology Co., Ltd., Wuhan, China) indicators, and tight junction proteins were detected by ZO-1 (GB111402-100, Wuhan Servicebio Technology Co., Ltd., Wuhan, China) and occludin (GB111401, Wuhan Servicebio Technology Co., Ltd., Wuhan, China) indicators. The positive expression and location of each indicator protein were observed under a microscope. After taking pictures, Image Pro Plus 6.0 image analysis was used to analyze each photo to obtain the positive cumulative optical density value (IOD) and pixel area (AREA) on each photo as well as to calculate the average optical density (AOD value). AOD = IOD/AREA, the larger the AOD value, the higher the positive expression level.

### 5.9. Western Blot Detection of the Effects of DON on the Expression of Inflammatory Cytokines in the Brain

The protein expression levels of inflammatory factors in the brain, IL-1β (ab315084, Abcam plc, Cambridge, UK), IL-6 (#12912, Cell Signaling Technology, Inc., Danvers, MA, USA), TNF-α (ab215188, Abcam plc, Cambridge, UK), IL-4 (bs-0581R, Bioss Inc., Woburn, MA, USA), and IL-10 (ab189392, Abcam plc, Cambridge, UK), were detected. Total protein was extracted from mouse brain tissue using RIPA lysis buffer (P0013B, Beyotime, Shanghai, China) supplemented with protease inhibitors. After SDS-PAGE separation, the membrane was transferred for 45–80 min. The bands were blocked with 5% skim milk powder at room temperature for 1 h, incubated with primary antibody at 4 °C overnight on a shaker, washed 3 times with 1 × TBST for 10 min each time, and incubated with secondary antibody (A0208, Shanghai Beyotime Biotechnology Co., Ltd. (Shanghai, China) at room temperature for 1.5 h. The dilution ratio of the secondary antibody was 1:500–1:2500. After the secondary antibody incubation was completed, the membrane was washed 3 times with 1 × TBST for 10 min each time. The membrane was then placed in a clean container and the prepared ECL color-developing solution (solution A: solution B = 1:1) was added and kept in the dark for 5 min. Bio-Rad gel imaging system was used to expose the membrane and collect images. Image-J(1.53r) software was used to analyze the grayscale value of the protein bands. After normalizing the data, the differences between the groups were calculated.

### 5.10. RT qPCR Detection of the Effects of DON on the mRNA Expression Levels of Glial Cell Markers, Neuronal Markers, Tight Junctions, and Inflammatory Factors in the Brain

Brain tissues snap-frozen in liquid nitrogen were ground into powder using a mortar and pestle, and total RNA was extracted from each sample using an animal total RNA isolation kit (Sagon Biotech, Shanghai, China) according to the manufacturer’s instructions. RNA concentration was measured using a UV spectrophotometer at 260 nm (Thermo Fisher Scientific, Waltham, MA, USA; Od260/280 ≈ 1.9–2.0). Specific primers for genes of brain glial markers (iba1 and GFAP), neuronal markers (NeuN and c-FOS), tight junction proteins (ZO-1 and occludin), pro-inflammatory factors (IL-1β, IL-6, TNF-α), and anti-inflammatory factors (IL-4 and IL-10) were designed using Oligo 7.0 software and synthesized by Shanghai Bio-Industry Technology Co., Ltd. (Shanghai, China). After reverse transcription of 2 μg RNA into 20 μL cDNA using the PrimeScrisp RT Kit (Takara, Tokyo, Japan), real-time quantitative PCR was performed using the CFX96 PCR Detection System (BioRad, Hercules, CA, USA) and SYBR Premix ExTaq (Takara Bio Inc., Shiga, Japan). The PCR conditions were as follows: 95 °C for 5 min, followed by 40 cycles of 95 °C for 15 s, annealing at 60 °C and 70 °C for 60 s, and extension for 25 s. The volume of each qRT-PCR reaction was 20 μL, which contained 10 μL TB Green TM Premix (Takara), 0.4 μL forward and reverse primers, 2 μL cDNA, and 7.5 μL DNase/rnase-free deionized water (Tiangen, Beijing, China). The gene primers used in this experiment are shown in Table 1. The relative expression was normalized to the internal reference GAPDH using the 2^−ΔΔCt^ method.

### 5.11. Statistical Analysis

SPSS 25.0 statistical analysis software was used to perform normality tests on experimental data. For experimental data that conform to normality, homogeneity of variance tests were performed. Two-way ANOVA tests were used for experimental data that conform to normality and homogeneity of variance. Independent sample *t*-tests were performed for each of the four “selected cases”, and the differences between the groups are shown in a bar chart. All experimental data are expressed as “Mean ± SD”, ns indicates no difference, “*” *p* < 0.05 indicates significant difference, and “**” *p* < 0.01 and “***” *p* < 0.001 indicates extremely significant difference.

## Figures and Tables

**Table 1:** Primers.

Gene Name	Primer	Sequence	Annealing Temp
*IL-1β*	F: TGGTGTGTGACGTTCCCATT R: TGTCGTTGCTTGGTTCTCCT	16,176	60/60
*IL-6*	F: TGTCGTTGCTTGGTTCTCCT R: TGCAAGTGCATCATCGTTGTTC	16,193	60/60
*TNF-α*	F: TCTTCTCATTCCTGCTTGTGG R: ATGAGAGGGAGGCCATTTG	21,926	60/60
*IL-4*	F:ACGGAGATGGATGTGCCAAAC R: AGCACCTTGGAAGCCCTACAGA	16,189	60/63
*IL-10*	F: GCCAGAGCCACATGCTCCTA R: GCCAGAGCCACATGCTCCTA	16,153	61/61
*ZO-1*	F: ACCAGATGTGGATTTACCCGTCA R: ACATCATTTCCACCAGCTAGTCG	21,872	61/60
*Occludin*	F: GGCAAGCGATCATACCCAGA R: GCTGCCTGAAGTCATCCACA	18,260	60/60
*c-FOS*	F: CGGGTTTCAACGCCGACTA R: TTGGCACTAGAGACGGACAGA	14,281	59/61
*GFAP*	F: CCTTCTGACACGGATTTGGT R: TAAGCTAGCCCTGGACATCG	14,580	58/60
*NeuN*	F: CCACCACTCTCTTGTCCGTT R: ATCAGCAGCGGCATAGACTC	52,897	60/60
*iba1*	F: GGATCTGCCGTCCAAAC R: GCATTCGCTTCAAGGACAR	11,629	54/53
*GAPDH*	F: CGACTTCAACAGCAACTCCCACTCTTCC R: TGGGTGGTCCAGGGTTTCTTACTCCTT	14,433	60/60

## Data Availability

The data presented in this study are available on request from the corresponding authors. The data are not publicly available due to privacy or ethical restrictions.
